# Reduced *Vglut2/Slc17a6* Gene Expression Levels throughout the Mouse Subthalamic Nucleus Cause Cell Loss and Structural Disorganization Followed by Increased Motor Activity and Decreased Sugar Consumption

**DOI:** 10.1523/ENEURO.0264-16.2016

**Published:** 2016-09-29

**Authors:** Nadine Schweizer, Thomas Viereckel, Casey J.A. Smith-Anttila, Karin Nordenankar, Emma Arvidsson, Souha Mahmoudi, André Zampera, Hanna Wärner Jonsson, Jonas Bergquist, Daniel Lévesque, Åsa Konradsson-Geuken, Malin Andersson, Sylvie Dumas, Åsa Wallén-Mackenzie

**Affiliations:** 1 Department of Organismal Biology, Uppsala University, SE-752 36 Uppsala, Sweden; 2 Department of Neuroscience, Uppsala University, SE-751 24 Uppsala, Sweden; 3 Faculty of Pharmacy, Université de Montréal, Montréal, QC H3T 1J4, Canada; 4 Oramacell, 75006 Paris, France; 5 Department of Pharmaceutical Biosciences, Uppsala University, SE-751 24 Uppsala, Sweden; 6 Department of Chemistry, BMC - Analytical Chemistry and Neurochemistry, Uppsala University, SE-751 24 Uppsala, Sweden

**Keywords:** dopamine, dynorphin, glutamate, rearing, reward, self-administration

## Abstract

The subthalamic nucleus (STN) plays a central role in motor, cognitive, and affective behavior. Deep brain stimulation (DBS) of the STN is the most common surgical intervention for advanced Parkinson’s disease (PD), and STN has lately gained attention as target for DBS in neuropsychiatric disorders, including obsessive compulsive disorder, eating disorders, and addiction. Animal studies using STN-DBS, lesioning, or inactivation of STN neurons have been used extensively alongside clinical studies to unravel the structural organization, circuitry, and function of the STN. Recent studies in rodent STN models have exposed different roles for STN neurons in reward-related functions. We have previously shown that the majority of STN neurons express the vesicular glutamate transporter 2 gene (*Vglut2/Slc17a6*) and that reduction of Vglut2 mRNA levels within the STN of mice [conditional knockout (cKO)] causes reduced postsynaptic activity and behavioral hyperlocomotion. The cKO mice showed less interest in fatty rewards, which motivated analysis of reward-response. The current results demonstrate decreased sugar consumption and strong rearing behavior, whereas biochemical analyses show altered dopaminergic and peptidergic activity in the striatum. The behavioral alterations were in fact correlated with opposite effects in the dorsal versus the ventral striatum. Significant cell loss and disorganization of the STN structure was identified, which likely accounts for the observed alterations. Rare genetic variants of the human *VGLUT2* gene exist, and this study shows that reduced *Vglut2/Slc17a6* gene expression levels exclusively within the STN of mice is sufficient to cause strong modifications in both the STN and the mesostriatal dopamine system.

## Visual Abstract

**Figure:**
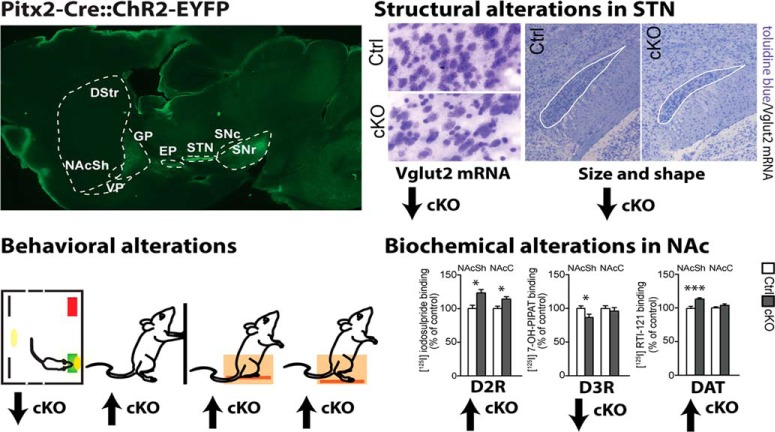

## Significance Statement

The subthalamic nucleus (STN) is the most common target in deep brain stimulation of advanced Parkinson’s disease, and it has recently been implicated in the reward circuit. Vesicular glutamate transporter 2 (VGLUT2) is the main vesicular transporter of glutamate in STN neurons, and rare genetic variants of the VGLUT2 gene exist in humans. Blunting *Vglut2/Slc17a6* gene expression levels throughout the extent of the mouse STN caused substantial cell loss in the STN, similar to that observed in pharmacologic lesion models. Mice became hyperactive but showed reduced sugar consumption. Opposite effects on motor versus reward behavior was correlated with opposite activity of dopamine parameters in the dorsal versus the ventral striatal system. This study thereby identifies an interaction between the STN and the dopamine reinforcement system.

## Introduction

The subthalamic nucleus (STN) is an excitatory structure that, via extensive glutamatergic projections, plays a central role in motor, limbic, and cognitive functions. Dysregulated excitatory output from the STN can be normalized by high-frequency deep brain stimulation (DBS). Clinically, STN-DBS is implemented for alleviation of motor dysfunction in in patients suffering from advanced Parkinson’s disease (PD; Benabid et al., 2009), and based on its role in limbic and cognitive functions, the STN has also been proposed as a putative target in DBS approaches to treat various neuropsychiatric disorders (Williams et al., 2010; Chabardès et al., 2013). In fact, STN-DBS has been tested with positive outcome in severe obsessive compulsive disorder (Mallet et al., 2008; Blomstedt et al., 2013).

In parallel to clinical studies of the STN, experimental animals have been used extensively to unravel the structural organization and circuitry of the STN as well as functional outcome upon STN intervention (Temel et al., 2005a; Baunez and Gubellini, 2010). In addition to STN-DBS experiments in animals, other models implement pharmacologic lesioning or inactivation to disturb STN function (Temel et al., 2005a; Pratt et al., 2012). Recently, a series of studies in rodent STN models have implicated the STN in the brain reward circuit alongside the classic mesoaccumbal reward pathway (Breysse et al., 2015). First, implementation of STN-DBS and STN lesioning were shown to elevate the motivation for food while reducing the motivation for cocaine (Baunez et al., 2002, 2005; Bezzina et al., 2008; Rouaud et al., 2010). Second, the presentation of either a positive (rewarding) or negative (aversive) stimulus (or cues conditioned to either stimulus) altered the firing pattern of neurons within the STN (Darbaky et al., 2005; Lardeux et al., 2009; Espinosa-Parrilla et al., 2013; Breysse et al., 2015). Third, although the question of the structural organization of the STN is the focus of debate between those favoring the hypothesis of the STN as a tripartite structure and those favoring an STN organization without strict anatomical boundaries (Temel et al., 2005b; Benarroch, 2008; Keuken et al., 2012; Alkemade and Forstmann, 2014), some recent studies have focused on identifying subpopulations of STN neurons. By electrophysiological profiling in rodents, distinct STN subpopulations were found that adapted their responses when the rewarding substance sucrose was exchanged for water (Lardeux et al., 2009, 2013) or an aversive substance, such as quinine (Breysse et al., 2015).

Thus, by adapting to various reward-related contexts, STN neurons form a cerebral hub which participates in even more aspects of motor, limbic and cognitive functions than previously thought. Similar to the etiology of PD, to which aberrant STN activity is believed to contribute (Rodriguez et al., 1998; Olanow and Tatton, 1999), dysfunctional STN activity may therefore contribute to reward dysfunction in patients. Clinically, a putative role in reward-related behavior is manifested by the recent proposals of the STN as a DBS target for treatment of addiction, eating disorders, and obesity (Pelloux and Baunez, 2013; Val-Laillet et al., 2015).

The multitude of roles ascribed to the STN further strengthens the case for solving how this compact nucleus is organized to encompass such a high level of complexity. Based on the excitatory nature of the STN, we recently addressed expression of the genes encoding the three different vesicular glutamate transporters (VGLUT1–3) in mice (Takamori, 2006; Liguz-Lecznar and Skangiel-Kramska, 2007) and could show that the vast majority of STN neurons express only the *Vglut2/Slc17a6* gene (Schweizer et al., 2014). Whereas a full knockout of the *Vglut2/Slc17a6* gene was previously demonstrated as lethal (Moechars et al., 2006; Wallén-Mackenzie et al., 2006), some rare genetic variants of the human *VGLUT2/SLC17A6* gene have been identified in schizophrenia and severe alcoholism (Flatscher-Bader et al., 2008; Shen et al., 2010; Comasco et al., 2014). By implementing conditional targeting of the *Vglut2/Slc17a6* gene in mice using the paired-like homeodomain 2 (*Pitx2)-Cre* driver to direct the targeting to the STN (Skidmore et al., 2008), Vglut2 mRNA levels were reduced by 40% exclusively within the STN structure (Schweizer et al., 2014). This reduction was shown to result in decreased postsynaptic excitatory activity in STN target neurons followed by strong behavioral hyperactivity, including decreased latency to movement (Schweizer et al., 2014). Despite hyperactivity, these STN-targeted mice did not perform more poorly in reward-baited memory and impulsivity tests, but they did take longer to collect the rewards. This finding led us to address the hypothesis that the *Pitx2/Vglut2*-coexpressing subpopulation of STN neurons is important for regulation of both motor activity and reward-related behavior.

## Materials and Methods

### Ethics statement

All mice used in the study were housed and produced as previously described (Schweizer et al., 2014) in accordance with the Swedish regulation guidelines (Animal Welfare Act SFS 1998:56) and European Union legislation (Convention ETS123 and Directive 2010/63/EU). Ethics approval was obtained from the Uppsala Animal Ethical Committee.

### Mice

The *Vglut2^f/f;Pitx2-Cre^* mouse line was produced by breeding *Pitx2-Cre* male mice (Skidmore et al., 2008) to floxed *Vglut2^f/f^* females (Wallén-Mackenzie et al., 2006) to generate *Vglut2^f/wt;Pitx2-Cre+^* male mice, which in turn were bred to *Vglut2^f/f^* females to generate conditional knockout (cKO; *Vglut2^f/f;Pitx2-Cre+^*) and control (*Vglut2^f/f;Pitx2-Cre-^*) mice. This allows for behavioral phenotyping and comparison between genotype groups because of identical genetic background (Crusio, 2004). Control *Vglut2^wt/wt;Pitx2-Cre+^* animals were used in the tracing experiment. All mice were kept on a hybrid background of C57BL/6J and Sv129.

### Operant self-administration of sucrose

**Figure 1.**
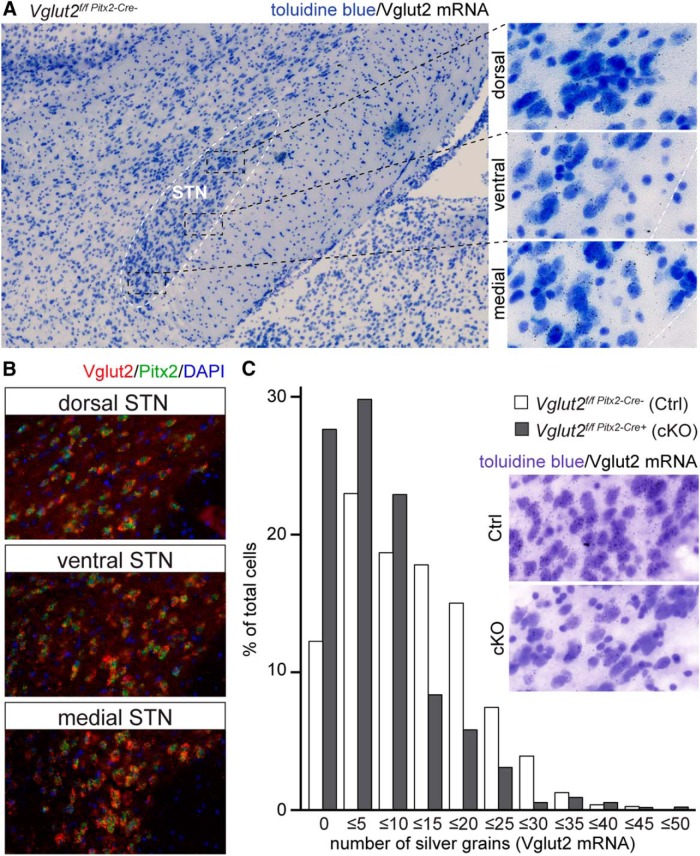
Strong overlap of Vglut2 and Pitx2 in the STN and reduction of Vglut2 mRNA in cKO mice. ***A***, Representative examples of cell morphology and distribution. Overview of the STN (left) as well as close-up images of the dorsal (right top), ventral (right center), and medial (right bottom). Cells are stained blue; small dots represent silver grains bound to Vglut2 mRNA. ***B***, Double fluorescent *in situ* hybridization for Vglut2 (red) and Pitx2 (green) as well as nuclear stain with DAPI (blue) in the dorsal (top), ventral (center), and medial (bottom) STN. ***C***, High-resolution *in situ* hybridization analysis of Vglut2 mRNA-based silver grain intensity shows a generally lowered expression of Vglut2 in the STN of cKO mice.

**Figure 2.**
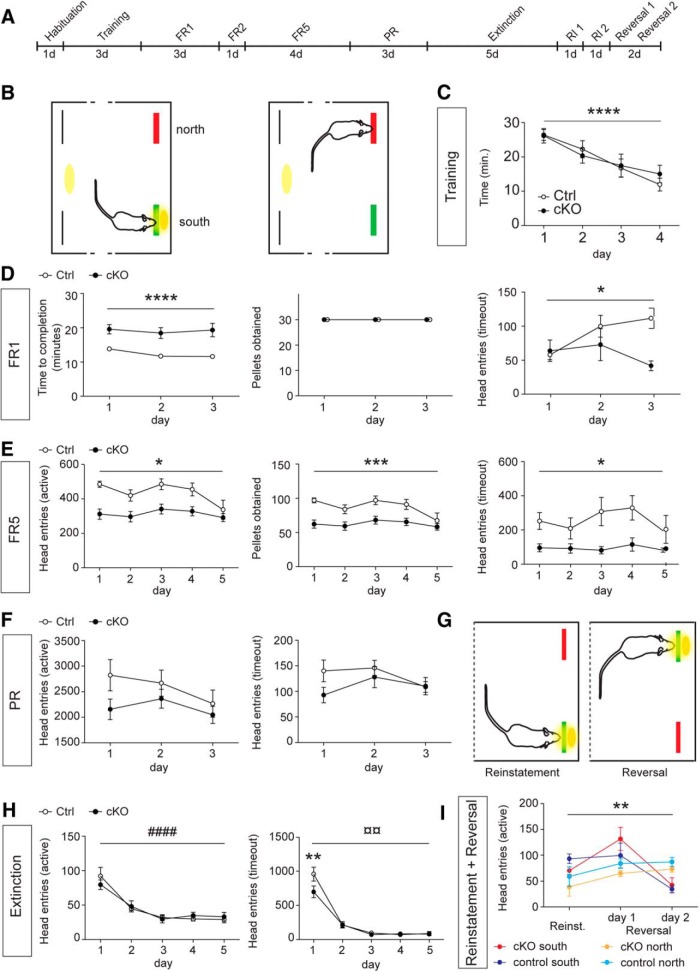
Decreased consumption of sugar in STN-VGLUT2 cKO mice. ***A***, Schematic self-administration schedule. Black lines indicate the number of days during which each paradigm was carried out. ***B***, Operant self-administration was carried out in modular self-administration boxes with two feeders, one of which delivered pellets upon head entry (south, green = active), and the other did not (north, red = inactive). Yellow: house light. Black bars, head entry holes measuring exploration with no feeder attached. When a mouse made a head entry at the active feeder, a sugar reward was delivered, and simultaneously, light and sound cues were presented to confirm the choice (left); a head entry at the inactive feeder produced neither reward delivery nor a light or sound cue (right). ***C***, During task learning, both controls (white circles) and cKOs (filled circles) decreased time to obtain the maximum of 30 sugar pellets. ***D***, During FR1, the time to consume 30 pellets was significantly higher for cKO (filled circles) compared with control (white circles; left panel). All mice were able to obtain the maximum of 30 pellets during the FR1 protocol (middle panel). The amount of head entries during the inactive time (timeout) decreased in cKO and increased in control (right). ***E***, During FR5, head entries during active time (left), the amount of pellets obtained (middle) and head entries during inactive time (right) were lower for cKO compared with controls. ***F***, During PR, no difference was seen in head entries during the active (left) or the inactive (timeout, right) time between control and cKO mice. ***G***, Cognitive ability testing. During reinstatement, the mice were presented to the original task after an extinction period (left). During reversal, the positions of the active and inactive feeders were switched (right). ***H***, To allow testing of the retention of the task (reinstatement), both cKO and control groups were put through extinction. For five consecutive days, the active feeder delivered both light and sound cues, but no sugar pellets. For both groups, the amount of head entries strongly decreased during both the active time (left) and timeout (right). ***I***, No differences between control and cKO mice were seen during reinstatement or reversal. Statistical analysis of the self-administration data was performed using repeated-measures ANOVA followed by a post hoc test with Bonferroni correction. ^#^ or *p ≤ 0.05; ^##^ or **p ≤ 0.01; ^###^ or ***p ≤ 0.001, ^####^ or **** *p* ≤ 0.0001. Hab., habituation; Rev., reversal; RI, reinstatement.

Twenty-three adult mice [13 males (seven cKO and six control) and 10 females (five cKO and five control)] were used to analyze the behavioral response to sugar in an operant self-administration setup (Med Associates, Fairfax, VT; Skinner, 1966; Sanchis-Segura and Spanagel, 2006). Source data for this experiment, summarized in Figure 2, are available at: http://goo.gl/slIH4u as Figure 2-1. An outline of the experiment is shown in Fig. 2*A*, and an illustration of the operant chamber, in Fig. 2*B*. Two days before starting and throughout the trial, the mice were under mild food restriction [3 g standard rodent chow (Lactamin, Lantmännen, Sweden) per mouse per day] and thereafter were weighed throughout the entire course of the experiment. Water was accessible *ad libitum*. The mice were allowed to habituate to the novel environment for one session before beginning training, and each mouse underwent one operant session per day. The mice were then trained to enter their head into a feeder equipped with a food receptacle and a photo beam sensor to obtain a 20-mg sucrose pellet (5TUL TestDiet). Each pellet delivery was followed by a 10-s timeout period, in which head entries were registered but no pellet could be obtained. Training (acquisition of the operant task) was performed on a fixed ratio (FR) 1 schedule (one pellet per head entry, maximum 30 pellets). Although a house light was continuously on, a cue light was lit only upon head entry into the feeder giving sugar, which also led to the presentation of a sound cue (Fig. 2*B*, left). Head entry into an inactive feeder did not result in cue or sugar presentation (Fig. 2*B*, right). During the timeout period, no cue light or sound cue was presented. FR1 training was followed by 3 days of testing on FR1 and then on FR5 to assess consummatory behavior. Before advancing the mice to an FR5 schedule (one pellet per five head entries), they were given one training day at FR2 to adjust to a task requiring multiple head entries. The mice were assessed in an FR5 schedule for 5 days, after which motivation to work for the sugar pellets was measured on a progressive ratio (PR) schedule, during which the amount of head entries required to obtain a pellet was successively increased by three per successful action. Next, extinction, reinstatement, and reversal of active and inactive feeders were used to assess task learning and cognitive flexibility.

During all trial days, the mice were left in the self-administration chambers until 30 sugar pellets were obtained (FR1, FR2, reinstatement, and reversal) or until trial time ended (FR5, PR, and extinction). Trial time was limited to 30 min for FR1, FR2, reinstatement, extinction, and reversal; 40 min for FR5; 90 min for PR, the maximum time possible for all animals to complete the PR task during the light-cycle of the day.

Statistical analysis was performed in GraphPad Prism 6 using repeated-measures ANOVA and Bonferroni post hoc test (GraphPad, San Diego, CA). A *p*-value ≤0.05 was considered significant (*p ≤ 0.05, ** *p* ≤ 0.01, *** *p* ≤ 0.001, **** *p* ≤ 0.0001).

### Analysis of rearing behavior

Thirty-one adult mice (cKO, *n* = 14; control, *n* = 17) were each placed in the center of a square Plexiglas arena (55 by 55 by 22 cm) and allowed to freely explore for 15 min. The mice were videorecorded, behavioral parameters were scored manually by an observer blinded to the genotype of the mouse, and the duration of each rearing type was analyzed using AniTracker software. Rearing behavior was subdivided into three types: wall rearing, an upright position with the fore-paws touching a vertical surface (Fig. 4*A*, left); seated rearing, a half-standing position using the tail for support (Fig. 4*B*, left); and free rearing, in which the mouse supports its weight freely on its hind legs without using its tail or forepaws (Fig. 4*C*, left), as previously described by Waddington et al. (2001). Data were analyzed using Mann–Whitney *U*-test (GraphPad Prism, GraphPad). A *p*-value ≤0.05 was considered significant (*p ≤ 0.05, ** *p* ≤ 0.01, *** *p* ≤ 0.001, **** *p* ≤ 0.0001).

### Dopamine receptor and transporter binding assay

Brains of cKO and control mice (cKO, *n* = 7; control, *n* = 7) were rapidly removed from the skull after cervical dislocation, cryosectioned, and processed for DA receptor and transporter autoradiography, as previously described (Mahmoudi et al., 2014; Schweizer et al., 2014). For DA D1 receptor (D1R) binding, the [^3^H]SCH23390 ligand was used. For D2R and D3R subtypes, [^125^I]iodosulpride and [^125^I]7-OH-PIPAT ligands were used, respectively. [^125^I]RTI-121 binding was used to measure dopamine transporter (DAT) density. Mann–Whitney *U*-test was performed for statistical analysis. A *p*-value ≤0.05 was considered significant (** *p* ≤ 0.01, *** *p* ≤ 0.001). All values are given as mean ± SEM, if not otherwise stated.

### Matrix-assisted laser desorption/ionization imaging mass spectrometry

Brains of cKO and control mice were cryosectioned at 12 µm, and sections encompassing the entopeduncular nucleus (EP) and globus pallidus (GP) were thaw-mounted with Pertex (HistoLab Products, Gothenburg, Sweden) on matrix-assisted laser desorption/ionization (MALDI) imaging–compatible conductive glass slides (Bruker Daltonics, Bremen, Germany). The slides were dried in a vacuum desiccator for 20 min and then stored at –20°C. Before analysis, the sections were thawed in a desiccator for 30 min and then washed for 10 s in (1) 70% ethanol, (2) 95% ethanol, and (3) 95% ethanol (Solveco Chemicals, Rosersburg, Sweden) and for 30 s in an acid wash (95% ethanol and 5% glacial acetic acid; Merck, Solna, Sweden). The washing steps and matrix deposition were earlier been developed for rat brain sections (Groseclose et al., 2007; Andersson et al., 2008).

Matrix was deposited by a piezoelectric-based Chemical Inkjet Printer (ChIP-1000; Shimadzu, Kyoto, Japan) to apply approximately 100-pl-sized drops of matrix in a grid across the tissue sections at a resolution of 200 µm (25 mg/ml DHB in 50% methanol, 10% ammonium acetate, 0.3% trifluoroacetic acid in water; five drops per pass and 25 passes).

MALDI imaging mass spectrometry was performed on an Ultraflex II MALDI TOF/TOF (Bruker Daltonics) in reflector mode. External calibration was performed with a standard peptide mix (Peptide Standard Calibration II; Bruker Daltonics). Baseline correction (convex hull) was performed for each spectrum and was exported as .dat files using FlexAnalysis (Bruker Daltonics). Spectra of the mass range from *m*/*z* 440 to 8660 were acquired from each matrix spot consisting of 600 laser shots in a 20-step random pattern. Total ion current normalization was performed on each spectrum, and data quality was assessed as previously described (Karlsson et al., 2014). A total of 5400 peaks with a signal-to-noise ratio >3 were selected, and peak borders were defined by binning analysis using mass spectrometry peak binning software pbin. The peak area was calculated for each peak using an R script developed in-house. Data are expressed as average peak area for 40–50 mass spectra collected from the nucleus accumbens (NAc) of each mouse (761 spectra in total collected from NAc of six cKO and three control mice). To specify the anatomic localization of peptides, photomicrographs of matrix deposits were coregistered with histologic staining of the same sections. After mass spectrometry acquisition, the matrix was removed with 95% ethanol and the mouse brain sections were stained with toluidine blue (Göteborgs Termometerfabrik, Göteborg, Sweden). FlexImaging 2.0 was used for visualization of peptide distribution. *F*-test ANOVA revealed unequal variance between groups, and the data were log-scaled for further analysis using Student’s *t*-test (two-tailed, α = 0.05, null hypothesis rejected at *p* < 0.05).

### *In situ* hybridization analyses

Radioactive *in situ* hybridization was performed as described in Schweizer et al. (2014) using antisense oligonucleotide probes for detection of Vglut2 mRNA. Two independent probes were used, one composed of a mix of three oligonucleotides [NM_080853.3: bases 13–47 (exon 1); bases 872–908 (exons 1 and 2); bases 3220–3254 (exon 12)] and one specific for exon 5 (bases 1432–1464). Pdyn was detected by a probe mix composed of a mix of three oligonucleotides (NM_018863.4: bases 1078–1112, 1694–1727, and 2309–2342). Images (Fujifilm BioImaging Analyzer BAS-5000, exposure time 1 month) corresponding to hybridized sections were exposed on film for mRNA expression analysis at a 25-µm resolution. For cellular mRNA expression analysis, slides were dipped in NTB emulsion and revealed after an exposition of 6 weeks; sections were counterstained with toluidine blue. For fluorescent detection, cryosections were air-dried, fixed in 4% paraformaldehyde and acetylated in 0.25% acetic anhydride/100 mM triethanolamine (pH=8). Sections were hybridized for 18 h at 65°C in 100 μl of formamide-buffer containing 1 μg/ml Vglut2-DIG-labeled riboprobe (NM_080853.3: bases 2315-3244) and 1 μg/ml Pitx2 fluorescein-labeled riboprobe (NM_001042504.1 :bases 792-1579). Sections were washed at 65°C with SSC buffers of decreasing strength, and blocked with 20% FBS and 1% blocking solution. Fluorescein epitopes were detected with HRP conjugated anti-fluorescein antibody at 1:1000 and revealed with TSA^™^ Kit (Perkin Elmer) using Biotin-tyramide at 1:75 followed by incubation with Neutravidin Oregon Green conjugate at 1:750. HRP-activity was stopped by incubation of sections in 0,1M glycine followed by a 3% H_2_O_2_.treatment. DIG epitopes were detected with alkaline phosphatase coupled anti-DIG fab fragments at 1:1000 and revealed with TSA^™^ Kit (Perkin Elmer) using Cy3 tyramide at 1:200. Nuclear staining was performed with DAPI. All slides were scanned on a 40× resolution NanoZoomer 2.0-HT (Hamamatsu, Japan). Cell size measurement, silver grain (mRNA expression), and cell counting were performed using ndp.view software (Hamamatsu). Histograms and scatter plots were produced with MATLAB plotting tools (Mathworks, Natick, MA).

### Analysis of projection patterns

*Vglut2^wt/wt;Pitx2-Cre+^* mice (*n* = 6) older than 8 weeks received 5 mg/kg carprofen before being anesthetized with isoflurane (0.5%–2%). Bilateral stereotactic injections of an adeno-associated virus carrying a Cre-dependent DNA construct of the *Channelrhodopsin-2* (*ChR2*) gene and the gene encoding the enhanced yellow fluorescent protein reporter (EYFP; 1 × 10^12^ vector genome/mL, UNC Vector Core Facility, Charlotte, NC) were carried out at anteroposterior, –1.9 from bregma, mediolateral, ±1.7 from midline. In each hemisphere, 250 nl were injected at dorsoventricular, –4.75 and –4.25, with a NanoFil syringe with a 35-gauge needle (World Precision Instruments, Sarasota, FL) at a rate of 100 nl/min. The needle was left in place for 10 min after the second injection and then slowly removed. Injected mice were kept in their home cage for at least 5 weeks to allow EYFP detection in synaptic terminals, then anesthetized with isoflurane and decapitated. The brain was removed and kept for 24 h in 4% formaldehyde solution. Coronal sections of 100 µm were prepared on a VT1200 Vibratome (Leica, Nussloch, Germany) and mounted in 90% glycerol. Images were taken on a Leica CTR 6000 microscope and analyzed with LAS AF Lite (Leica).

### Histologic analysis of STN structure

Three-dimensional reconstruction was obtained with MATLAB 3D plotting tools. Sixteen sections for knockout mice and 27 for wild-type mice were used to draw contours of the STN. These images were adjusted so that adjacent sections best fitted the real spatial position. We applied 3D cubic spline interpolation for missing sections and standard box smoothing. Different transparency, light shedding, and shading options enabled visualization of the 3D shape of the STN.

## Results

### Vglut2 and Pitx2 mRNAs overlap and are both distributed over the entire STN

The STN in both primates and rodents has been suggested to constitute a tripartite structure composed of a dorsal motor aspect with efferent projections to the globus pallidus interna (GPi; in mice known as the entopeduncular nucleus) and externa (GPe; in mice commonly referred to only as globus pallidus); a ventral aspect that, via projections to the substantia nigra pars reticulata (SNr), regulates associative behavior; and a medial aspect that communicates with the limbic system via the ventral pallidum (VP; Kita and Kitai, 1987; Groenewegen and Berendse, 1990; Benarroch, 2008; Alkemade and Forstmann, 2014). This model is based on projection patterns and functional outcome, but no genetic marker has been identified that distinguishes between these three aspects of the STN.

The *Vglut2/Slc17a6* gene encoding the synaptic protein VGLUT2 is strongly expressed (mRNA) within the cytoplasm of neurons located in the STN in both rodents and primates (Hisano, 2003; Barroso-Chinea et al., 2007; Rico et al., 2010) representing the major glutamatergic population within the STN in mice (Schweizer et al., 2014). For this reason, we wished to assess the distribution pattern of *Vglut2/Slc17a6*-expressing neurons within mouse STN to analyze whether these distribute differently within the dorsal, ventral, and medial aspects of the STN. High-resolution radioactive Vglut2 mRNA-selective *in situ* hybridization analysis was performed to enable visualization and subsequent quantification of Vglut2 mRNA. Vglut2 mRNA, detected as silver grains, was readily visible across the entire extent of the STN (Fig. 1*A*, left). Whereas Vglut2 mRNA was present throughout all aspects of the STN, the level of expression was visibly higher in the medial and ventral parts. Most cells contained Vglut2 mRNA, but cells of different sizes appeared throughout the STN (Fig. 1*A*, right).

Further, because the *Pitx2* gene has been shown to be highly expressed in the STN (Martin et al., 2004), we next used double fluorescent *in situ* hybridization to analyze the distribution of Pitx2 mRNA, as well as the overlap between Vglut2 and Pitx2 mRNA on a cellular level. A near-100% overlap between these two mRNAs was observed throughout all aspects of the STN (Fig. 1*B*). The distribution pattern of both Vglut2 and Pitx2 mRNA was important to establish because, as discussed above, we use a *Pitx2-Cre* transgenic mouse line (Skidmore et al., 2008) as a tool to enable targeted deletion of *Vglut2/Slc17a6* expression within the STN (Schweizer et al., 2014). However, we had not previously addressed the possibility of subregional targeting within this structure. Our previous analysis showed that the *Vglut2^f/f;Pitx2-Cre+^* cKO mice and their control littermates (*Vglut2^f/f;Pitx2-Cre-^*) as expected had similar levels of Pitx2 mRNA in the STN. However, *Vglut2/Slc17a6* expression levels were blunted in the cKO STN, so the amount of cells with low-level expression was increased at the expense of cells with high-level expression, leaving a 40% decrease in Vglut2 mRNA (Schweizer et al., 2014).

Having now ensured that *Pitx2* and *Vglut2/Slc17a6* expressions were overlapping and distributed all over the STN, we next quantified the amount of Vglut2 mRNA by counting the number of silver grains in 550 STN neurons of cKO mice and 792 neurons of control mice. Approximately 12% of all the counted STN neurons in the control mice lacked any detectable Vglut2 mRNA, whereas approximately 88% of STN neurons showed a wide range of Vglut2 mRNA expression levels (Fig. 1*C*). The number of cells expressing no detectable Vglut2 mRNA at all was more than double throughout the STN structure in the cKO compared to control (28% vs. 12%). Further, the number of low-level Vglut2 mRNA levels (1–10 silver grains/cell) was higher in the cKO than control STN, and more control than cKO STN cells showed higher Vglut2 mRNA levels (11 or more silver grains/cell).

These histologic analyses show that in the mouse, Pitx2 and Vglut2 mRNAs are highly overlapping (near 100%) throughout the extent of the STN and thus do not represent any specific aspect of the STN. Moreover, in control mice, almost 90% of all STN neurons express the *Vglut2/Slc17a6* gene, whereas the majority of cKO neurons in the STN contain no or low levels of Vglut2 mRNA. Thus, the current results demonstrate that *Vglut2/Slc17a6* expression levels are severely blunted throughout the entire STN structure of *Vglut2^f/f;Pitx2-Cre+^* (cKO) mice.

### Normal reward-associated learning, memory, and cognitive flexibility but lower sugar consumption when Vglut2/Slc17a6 expression is blunted within the STN

Hyperactive individuals (mice as well as humans), can be lean without having any kind of deficiency in food consumption. Therefore, the slightly smaller size of the hyperactive cKO mice did not appear abnormal at first, but because a lower rate in collecting high-fat food pellets in the baited radial arm maze and in the delay discounting test was measured (Schweizer et al., 2014), a more thorough analysis of feeding behavior was undertaken.

Generally, decreased consumption of fatty food might reflect decreased eating overall or a lack of consumption of palatable eatables specifically, the latter of which could reflect a deficiency in the mesostriatal reinforcement system (Everitt and Robbins, 2005), also known as the brain reward system. To differentiate between these two possibilities, voluntary consumption of high-sucrose food, another palatable eatable, was assessed in an operant self-administration paradigm (Skinner, 1966) under mild food restriction [Fig. 2*A* (schematic presentation of the entire experiment), 2*B* (illustration of the operant chamber)]. The results of this analysis showed that no difference between control and cKO groups could be observed in task acquisition (training), as both groups learned equally well to collect the maximum of 30 pellets and decreased the time for task completion during training (change over time: *p* ≤ 0.0001; *F* = 13.16, Df = 3; Fig. 2*C*). Both groups were able to reliably collect 30 pellets on three consecutive days on the FR1 schedule (Fig. 2*D*, left and middle). However, the time to complete the FR1 task was significantly increased for cKO mice compared with controls (variation between genotypes: *p* ≤ 0.0001; *F* = 23.58, Df = 1; Fig. 2*E*, left). Furthermore, the control mice continued to increase the number of head entries during timeout over the trial, whereas the number of head entries of cKO mice declined (time × genotype: *p* = 0.00113; *F* = 5.024, Df = 2; Fig. 2*D*, right). During FR5, the control mice showed a higher number of head entries than cKO mice when a pellet could be obtained (time × genotype: *p* = 0.0336; *F* = 2.802, Df = 4; variation between genotypes: *p* = 0.0076; *F* = 9.476; Df = 1; Fig. 2*E*, left), thus leading to a higher consumption of sugar pellets (time × genotype: *p* = 0.0336; *F* = 2.802; Df = 4; variation between genotypes: *p* = 0.0076; *F* = 9.476; Df = 1; Fig. 2*E*, middle). In addition, cKO mice displayed decreased seeking for sugar during timeout in the FR5 paradigm (variation between genotypes: *p* = 0.0175; *F* = 7.012; Df = 1; Fig. 2*E*, right).

Whereas FR5 is used to measure sugar consumption rate, the PR paradigm has been described as a measure for the motivational aspect of consumption (Sanchis-Segura and Spanagel, 2006). During PR, the number of head entries when the feeder was active (time × genotype: *p* = 0.3776; *F* = 0.9992, Df = 2) or during timeout (time × genotype: *p* = 0.1506; *F* = 1.986, Df = 2) was similar between cKO and controls (Fig. 2*F*). A breaking point was not observed in either group. During the extinction phase, both groups decreased their number of head entries at a similar pace (change over time: *p* ≤ 0.0001; *F* = 45.24, Df = 4; variation between genotypes: *p* = 0.9457; *F* = 0.0049; Df = 1; Fig. 2*H*, left). Head entries during timeout decreased as well (time × genotype: *p* = 0.0085; *F* = 4.105, Df = 4; change over time: *p* ≤ 0.0001; *F* = 130.2, Df = 4; variation between genotypes: *p* = 0.2763; *F* = 1.365, Df = 1) with head entries in cKO only significantly lower on the first day (post hoc test with Bonferroni correction, *p* < 0.01; *t* = 3.736) but not the other days (Fig. 2*H*, right). Upon reinstatement of the operant task, control and cKO groups showed no difference in level of activity at the active feeder (*p* > 0.05) and were also equally able to switch sides upon reversal of the active and inactive feeders (time × genotype: *p* = 0.0015; *F* = 4.005, Df = 6; change over time: *p* = 0.0014; *F* = 7.172, Df = 2; Fig. 2*I*). Throughout the experiment, activity on the inactive feeder was low in both the control and cKO groups (source data Figure 2-1, available at http://goo.gl/slIH4u).

Analysis of operant sugar consumption behavior thus demonstrates that although the cKO mice displayed normal reward-related learning, motivation, memory, and ability for task-switching, their consummatory rate of sugar eatables was significantly reduced.

### Lower weight but normal refeeding after food restriction

**Figure 3.**
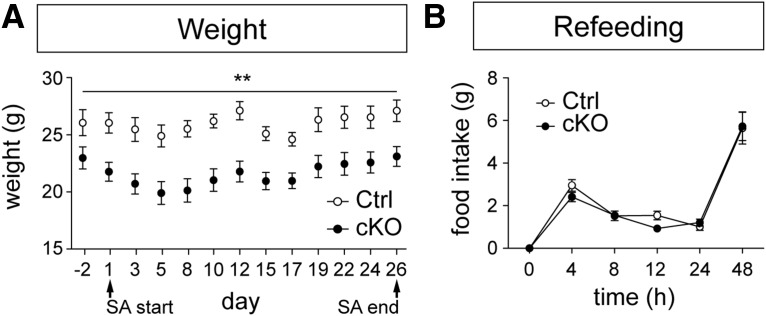
The average weight of STN-VGLUT2 cKO mice is decreased, whereas home cage food consumption is unaltered. ***A***, Control mice (white circles) had a higher body weight than cKO mice (filled circles) over the course of the experiment. ***B***, Refeeding after self-administration was not significantly different between controls and cKOs. Statistical analysis of weight and food intake data were analyzed using repeated-measures ANOVA followed by a post hoc test with Bonferroni correction. *p ≤ 0.05; **p ≤ 0.001.

During the whole self-administration regimen, mice were weighed daily to control for body weight fluctuations. This procedure showed that the cKO mice weighed less than controls throughout the trial but that cKO and control mice followed the same overall shifting in weights between days (time × genotype: *p* = 0.0011; *F* = 2.860, Df = 12; Fig. 3*A*). Upon completion of the self-administration task, all mice were allowed to feed freely in their home cage environment (refeeding). Despite weight differences, both control and cKO mice consumed similar amounts of standard rodent chow during refeeding (time × genotype: *p* = 0.6323; *F* = 0.6911, Df = 5; Fig. 3*B*).

Because the cKO mice consumed normal amounts of standard chow during refeeding after the operant task, the results suggest that the observed effect on sugar consumption is specific to sweet food and that the leaner shape of the cKO mice before food-restriction rather reflects their hyperactivity than a deficiency in standard chow feeding.

### Significant increase in seated and free rearing corroborates elevated motor activity

**Figure 4.**
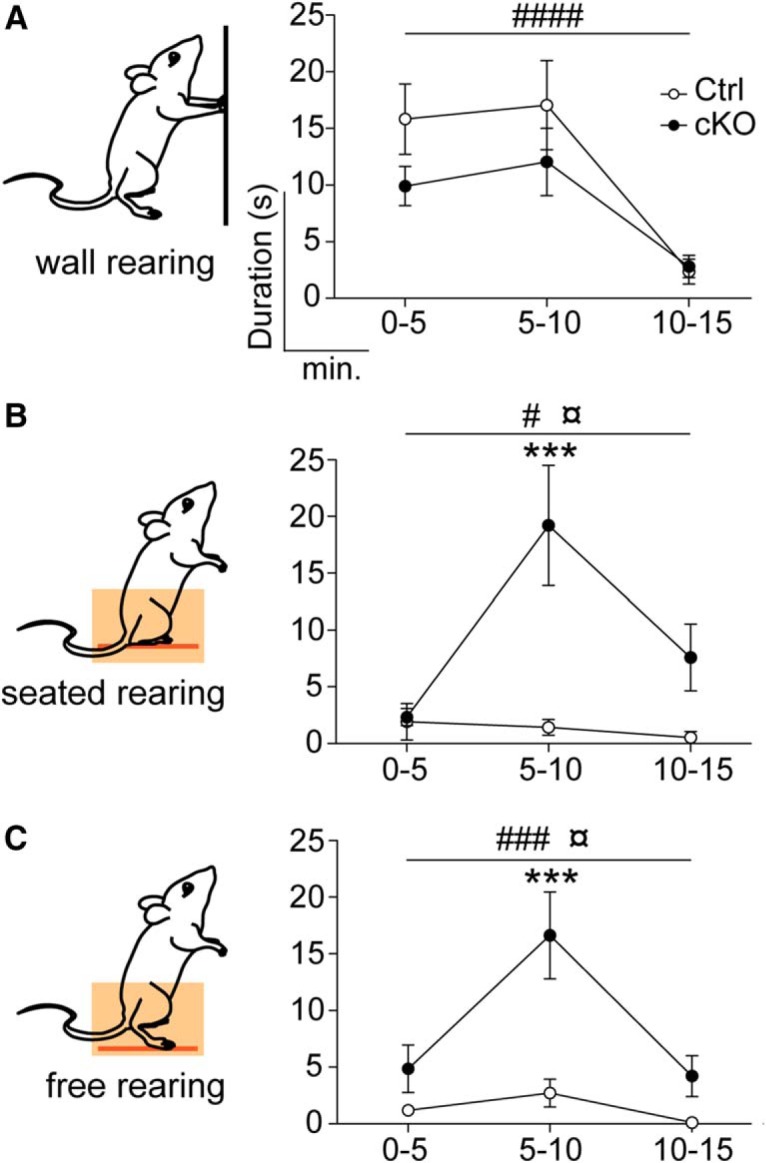
Increased seated and free rearing in STN-VGLUT2 cKO mice. Three rearing types were observed in STN-VGLUT2 cKO mice: wall rearing, where the mouse takes support against a vertical surface (***A***, left); seated rearing, where the mouse supports itself on the tail base (***B***, left); and free rearing, where the mouse stretches its hind legs and uses no other support (***C***, left). All rearing types were scored in an open field arena. Both groups were habituated to the environment (# marks the change over time). cKOs show increased seated (***B***) and free (***C***) rearing throughout the session (¤ marks the difference between genotypes and * marks the difference at a certain time point between genotypes). Rearing data was analyzed by repeated-measures ANOVA and post hoc test with Bonferroni correction. *p ≤ 0.05; **p ≤ 0.001; ***p ≤ 0.0001.

*Vglut2^f/f;Pitx2-Cre+^* cKO mice are both vertically and horizontally hyperactive (Schweizer et al., 2014). After showing that they consume less sugar, we wished to know more about their motor behavior. Rearing behavior differs from locomotion, as it does not serve to move the mouse forward, but instead signifies a sensing of the environment, commonly interpreted as an exploratory behavior. Further, rearing displayed in a repetitive format is commonly interpreted as a form of stereotypy in rodent models of various neuropsychiatric disorders (Kim et al., 2016). It has also been shown that the presentation of rearing behavior in mice can appear in different ways depending on body posture and the positioning of the paws and tail (Waddington et al., 2001). To explore the type of rearing accentuated in the *Vglut2^f/f;Pitx2-Cre+^* cKO mice, we analyzed it in detail by subcategorizing rearing into three subtypes; wall rearing (Fig. 4*A*, left), seated rearing (Fig. 4*B*, left), and free rearing (Fig. 4*C*, left), as previously described (Waddington et al., 2001). By quantifying the duration of each of the types of rearing, no significant difference in wall rearing was found between control and cKO mice (variation between genotypes: *p* = 0.2577; *F* = 1.441, Df = 1; Fig. 4*A*, right). In contrast, both seated rearing (interaction: *p* = 0.0127; *F* = 5.625, Df = 2; Fig. 4*B*, right) and free rearing (*p* = 0.0081; *F* = 6.366, Df = 2; Fig. 4*C*, right), the two more complex forms of rearing, were significantly overrepresented in the *Vglut2^f/f;Pitx2-Cre+^* cKO group of mice (Fig. 4).

Summarizing the behavioral findings, the results demonstrate that mice lacking normal expression levels of the *Vglut2/Slc17a6* gene in the STN display severely elevated motor activity, including advanced subtypes of rearing barely detected in control mice, and blunted reward consumption. These findings suggest an overactivity of the motor system at the expense of the reward system, a finding that next motivated analysis of the mesostriatal dopamine (DA) system.

### Significant alterations in DA receptor and DA transporter capacity

DA neurotransmission in the dorsal striatum (DStr), mediated by the nigrostriatal DA neurons located in the substantia nigra pars compacta (SNc), is strongly correlated with locomotion and rearing, whereas DA transmission in the NAc, the ventral aspect of the striatum, is primarily mediated by DA neurons of the ventral tegmental area and is triggered by palatable food and drugs of abuse (Björklund and Dunnett, 2007). The reduced interest in palatable food rewards alongside the elevated motor behavior thus suggested an opposing circuitry effect by the targeted deletion of the *Vglut2/Slc17a6* gene in the STN on dorsal and ventral striatal DA signaling. Indeed, in our previous analyses, we showed that the nigrostriatal DA system is overactive, with increased levels of DA release and reduced DA clearance due to reduced levels of the DAT in the DStr, whereas no alteration in terms of DA receptor availability was detected (Schweizer et al., 2014).

**Figure 5.**
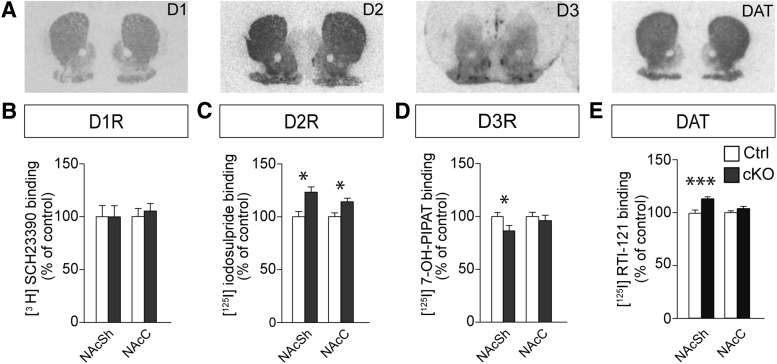
The availability of dopamine receptor D2 and D3 as well as DAT binding sites in the ventral striatum is regulated by Vglut2 reduction in the STN. ***A***, Representative examples of serial coronal striatal sections analyzed for DAT, D1R, D2R, and D3R (from top to bottom). ***B***, Comparison of specific binding capacity levels expressed as percentage of control for DAT-specific [^125^I]RTI binding, D1R-specific [^3^H]SCH23390, D2R-specific [^125^I]iodosulpride, and D3R-specific [^125^I]7-OH-PIPAT binding in control and cKO mice. DAT binding was unaltered in the NAcC and upregulated in the NAcSh of cKO mice (left). The amount of D1 receptor binding sites (middle left) in both NAcC and NAcSh remained unaltered, whereas more D2 receptor binding sites were measured in both NAcC and NAcSh (middle right) and fewer D3 receptor binding sites were available in NAcSh (right). Data analyzed by Mann–Whitney *U*-test. *p ≤ 0.05.

Having now unraveled an effect of the *Vglut2* targeting of the STN on consumption of natural rewards, we focused on addressing a possible contribution of the NAc. The levels of DA receptors are important for the hedonic impact of drugs of abuse and palatable food (Volkow et al., 1999; Wenzel and Cheer, 2014). We therefore implemented receptor autoradiography of [^3^H]SCH23390, [^125^I]iodosulpride, and [^125^I]7-OH-PIPAT binding (Mahmoudi et al., 2014) to measure the density of DA receptor D1R, D2R, and D3R subtypes, respectively (Fig. 5*A*). No difference between control and cKO mice was detected for D1R in either the core or shell subregions of the NAc (NAcC and NAcSh, respectively; Fig. 5*B*). However, for both D2R and D3R, ligand binding was altered in the NAcSh. D2R binding was elevated above control levels in the cKO brain, whereas D3R ligand binding was decreased (Fig. 5*C*, *D*). In addition, D2R binding was above control levels in the cKO NAcC (Fig. 5*D*). Next, we assessed DAT capacity using [^125^I]RTI-121 binding. [^125^I]RTI-121 binding was significantly increased in the NAcSh of the cKO mice compared to controls (Fig. 5*E*).

These findings point to different, and even opposite, modifications in the dorsal versus the ventral striatal DA systems upon the targeted deletion of *Vglut2* in the STN, such that the NAcSh of the cKO mice have altered D2R and D3R availability as well as increased DAT capacity, as opposed to unaltered receptor state and decreased DAT capacity in the DStr (Schweizer et al., 2014). The higher activity of the dorsal over the ventral striatal DA system likely accounts for the observed strengthening of motor behavior and weakening of the natural reward-related behavior; with focus on the reward system, the NAc was therefore analyzed in more detail.

### Altered levels of dynorphin neuropeptide, but not gene expression, in the NAc

**Figure 6.**
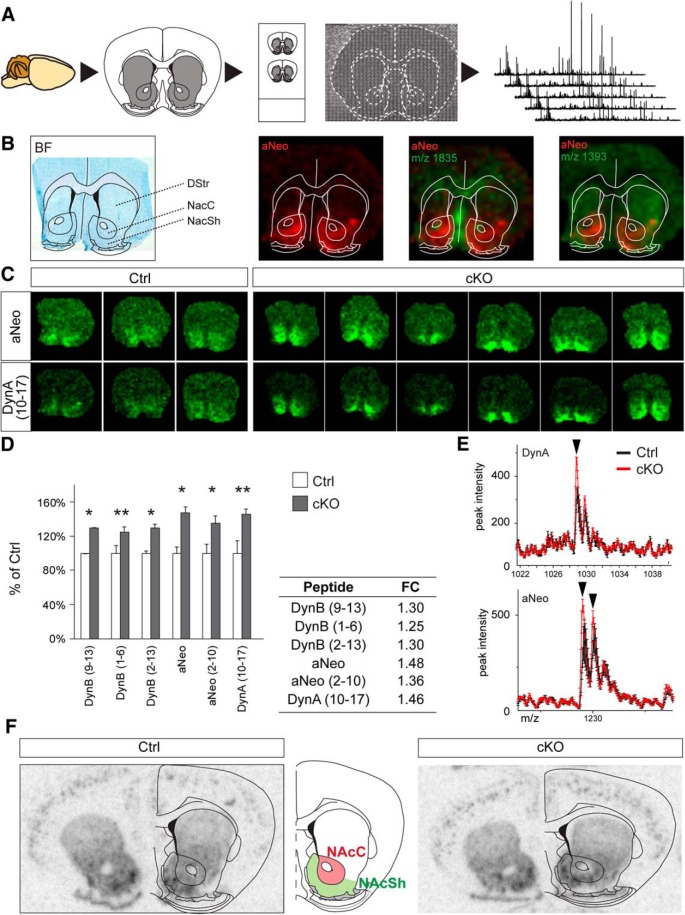
Elevated levels of dynorphin peptides in the NAc of STN-VGLUT2 cKO mice. ***A***, MALDI analysis was performed on cryosections at the level of the DStr and NAc. Each slice was coated with droplets of matrix for ionization of the peptide fragments and analyzed by MALDI time-of-flight. ***B***, A bright-field picture of the cresyl violet–stained section analyzed for aNeo was used to determine the localization of DStr and NAc (black outlines). Representative examples of aNeo (depicted in red) in cKO. Overlays with peptide fragment *m*/*z* 1835 and 1393 (middle and right, depicted in green) visualized the fiber bundles used as landmarks for the anatomical localization of the NAc. ***C***, Representative examples of slices used for MALDI imaging in control (left) and cKO (right). aNeo and DynA peptide levels were significantly elevated in cKO and showed a more restricted localization to the NAc. ***D***, Fluorescence intensity of different peptides measured by MALDI imaging, shown as percentage of control (fold change, see table). ***E***, Example peaks of DynA (top) and aNeo (bottom) from MALDI analysis. ***F***, Oligo in situ hybridization analysis of Dyn expression in the forebrain of control (left) and cKO (right) mice. FC: fold change; numbers in brackets indicate which amino acids constitute each peptide fragment. *p ≤ 0.05 and **p ≤ 0.01; error given as SEM.

To enable analysis of the molecular constitution within the neurons of the NAc and to assay for peptide alterations within this region of cKO and control mice, we implemented MALDI imaging as previously described [Hanrieder et al., 2011; Ljungdahl et al., 2011; Fig. 6*A* (schematic illustration of the procedure)]. The unknown peptides with *m*/*z* 1835 and 1393 were used to visualize white matter fiber tracts and the striatum, respectively, serving as landmarks for localization of the NAc (Fig. 6*B*).

The majority of the more than 100 peptides detected in the MALDI imaging were not differentially expressed in cKO and control animals. However, 19 peaks were significantly downregulated and 48 upregulated in cKO mice. Among the upregulated peptides, the strong changes in the family of dynorphin (Dyn) neuropeptides were most striking. Ion images for the Dyn peptides DynA and α-neoendorphin (aNeo) revealed visibly elevated Dyn levels in the NAc (Fig. 6*B*). Ion images obtained from cKO mice exhibited 25% to 50% higher levels of Dyn and aNeo peptides compared with control mice. These alterations were mainly localized to the NAc (Fig. 6*C*). Dyn peptides including DynB (9–13; *p* = 0.0290), DynB (1–6; *p* = 0.0087), DynB (2–13; *p* = 0.0334), aNeo (*p* = 0.0196 for first and *p* = 0.0352 for second isotope), aNeo (2–10; *p* = 0.0289), and DynA (*p* = 0.0021) were upregulated in the NAc of cKO compared with control brains (Fig 6*D*). The Dyn neuropeptides are products generated by prohormone convertase cleavage of the prodynorphin (Pdyn) propeptide (Berman et al., 2000; Yakovleva et al., 2006; Orduna and Beaudry, 2016). Dyn peptides can represent either afferent or local projections, so expression of the *Pdyn* gene was next analyzed at the mRNA level by in situ hybridization in the NAc. No difference in expression between control and cKO could be detected, suggesting that the regulation of the Pdyn peptide products does not occur at mRNA but at the peptide level (Fig. 6*F*).

Taken together with the results from the DA receptor and DAT binding shown above, these findings reveal that the targeted reduction of the *Vglut2/Slc17a6* gene expression levels within the STN had a strong impact on the mesostriatal DA reward system, and we sought to explore this correlation further.

### *Pitx2*-*Cre*–expressing STN neurons show projection patterns unique to EP/GP and SNr

**Figure 7.**
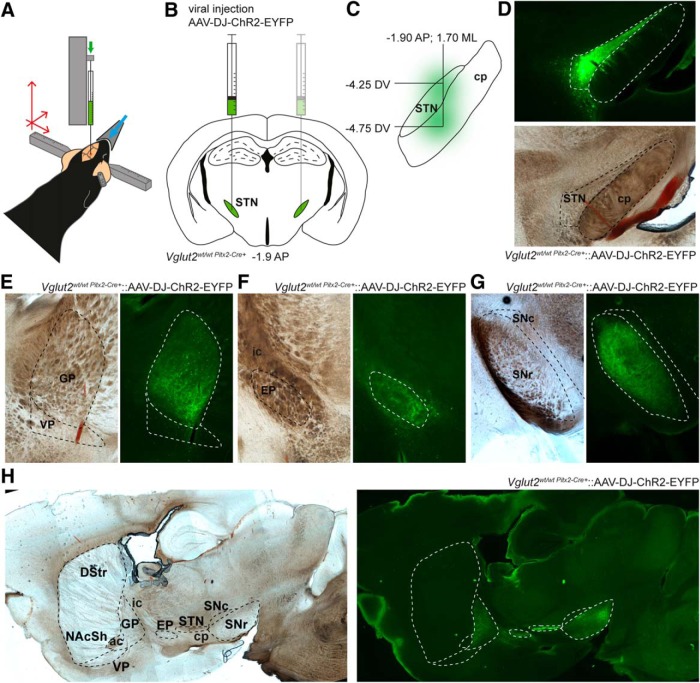
Optogenetic tracing of Vglut2-Pitx2 coexpressing neurons revealed a projection pattern restricted to the EP and SNr. ***A–C***, Schematic illustrations of injection procedure. ***A***, Mice were stereotactically injected with AAV-ChR2-EYFP into the STN; ***B***, The injection was performed bilaterally but one side at a time to infect the left- and righthand side STN at equal levels; ***C***, Two close coordinates were used for each STN to allow even spread of the virus (–4.25 and –4.75 dorsoventricular). ***D***, Histologic results of injections. AAV-ChR2-EYFP–positive cell bodies in the STN. ***E***, AAV-ChR2-EYFP–positive fibers projecting to EP (left), GP (middle), and SNr (right). Upper row, EYFP expression; bottom row, bright-field photographs of the same area. PSTN: parasubthalamic nucleus.

The STN has been reported to send a multitude of efferents to the basal ganglia and their associated structures, including the GPi/EP, GPe/GP, SNr, VP, pedunculopontine nucleus, SNc, and basolateral amygdala (Kita and Kitai, 1987; Parent and Hazrati, 1995; Parent et al., 2000; Sato et al., 2000; Benarroch, 2008; Degos et al., 2008; Watabe-Uchida et al., 2012; Hachem-Delaunay et al., 2015). However, to our knowledge, no direct innervation from the STN to either the NAc or DStr has so far been identified. We already confirmed a strongly reduced glutamatergic activity by implementing patch-clamp recordings upon both electrical and optogenetic stimulations in postsynaptic neurons in the main STN target areas EP and SNr (Schweizer et al., 2014). We now aimed to establish whether there is a projection from *Pitx2/Vglut2* coexpressing STN neurons directly to the NAc, which could explain the strong effects observed in this region of the cKO mice. To selectively trace the efferent projections and thereby pinpoint their target areas, an adeno-associated virus carrying a Cre-dependent DNA construct *ChR2* and the gene encoding EYFP was stereotactically injected bilaterally into the STN of control *Vglut2^wt/wt;Pitx2-Cre+^* mice (Fig. 7*A*, *B*). Virus was injected in two different dorsal-ventral stereotactic STN coordinates to ensure expression throughout the length of the STN (Fig. 7*C*). Histologic analysis revealed absence of EYFP in structures surrounding the STN, whereas ample EYFP-positive cell bodies were detected throughout the extent of the STN, confirming both successful expression of the *ChR2-EYFP* construct exclusively in the *Pitx2-Cre*–expressing neurons and the observation described above, that *Pitx2/Vglut2* coexpressing cells are distributed uniformly over this structure (Fig. 7*D*). The EP, GP, and SNr were verified as targets of the *Pitx2-Cre*–expressing STN neurons by EYFP localization to fibers throughout these areas (Fig. 7*E–G*). However, no EYFP-positive innervation at all could be detected in either the NAc or the DStr (Fig. 7*H*). Further, EYFP could not be detected in any of the other target areas described for the STN in different species: SNc, VP, basolateral amygdala, or pedunculopontine nucleus (Fig. 7*H*).

The results of these tracing studies thereby show that the alterations observed on the DA parameters in the NAc are most likely mediated via the already established target areas of the *Pitx2/Vglut2* coexpressing neurons, EP, GP, or SNr, and not via a direct projection from the STN to the NAc. To further explore how the *Vglut2* targeting of the STN might have caused the striatal alterations observed, we next sought to explore whether the targeted blunting of *Vglut2/Slc17a6* gene expression might have caused any alterations in the structure of the STN that might contribute to the dopaminergic phenotype observed.

### Cell loss as well as altered structural size and shape of the STN

**Figure 8.**
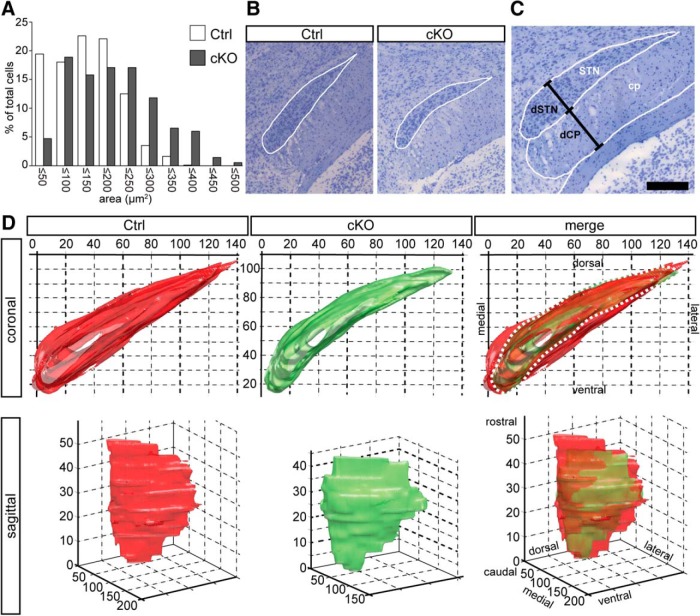
Vglut2 reduction leads to cell loss and reduction in size of STN. ***A***, Cell size is increased in cKO (black bars) mice compared with controls (white bars). The cKO mice show a much wider spread of cell size than controls. ***B***, Representative pictures of STN morphology in cKO and control. The STN of cKO mice appears slimmer compared with controls. The white outlines were used for 2D and 3D analysis of STN morphology. ***C***, Two-dimensional analysis of STN morphology. The size of the STN was assessed by measuring the diameter of the STN at its widest point and comparing it to the diameter of the cerebral peduncle (cp) and the total diameter of both structures at the same point. ***D***, Representative example of a control (left, red) and a cKO STN (middle, green), and their overlay (right). Both diameter (white dotted line) and volume of the cKO STN is reduced compared with the control. Three-dimensional reconstruction was obtained with MATLAB from 16 and 27 images of a cKO and a control, respectively.

A previous study of dopamine-glutamate coreleasing neurons in the midbrain has shown that expression of the *Vglut2/Slc17a6* gene is important for maintaining cell density (Fortin et al., 2012). During visual inspection of cryosectioned brains, we detected a difference in the shape of the STN between cKO and control mice; for this reason, we decided to quantify cell density and measure the size and shape of the STN. Toluidine blue contrast staining of the serially sectioned control and cKO mice used to quantify Vglut2 mRNA described above enabled quantification of cell number and cell size (area) in the same material. This analysis revealed a reduction in total number of cells by 39% in cKO compared with control. Further, more STN neurons in the cKO than control mice had larger cell area (201–350 µm^2^), leading to a right-sided shift in the histogram presenting cell area as a function of percentage of total cells (Fig. 8*A*). It was also evident that within the STN of cKO mice, there are neurons of a larger size range (351–500 µm^2^) than observed at all in the control STN.

We next analyzed whether the overall structure of the STN in the cKO mice had been altered as a consequence of this cell loss. For this task, 2D and 3D reconstruction analyses were performed in parallel. For 2D analysis, anatomical outlines of the STN were made on each section microscope photograph, and linear measurements were made at the widest part of the STN and the adjacent cerebral peduncle (Fig. 8*B*, *C*). These analyses revealed that the STN was slimmer in the cKO than in the control animals, whereas the cerebral peduncle was wider, thus pointing to shrinkage of the STN in the cKO mice. The STN outlines were subsequently used to establish 3D overlay pictures of cKO and control STNs from both coronal and sagittal views, both of which readily exposed the smaller size of the STN in the cKO mice (Fig. 8*D*). Further, the ventral and dorsomedial aspects of the STN were exposed as the regions most affected by the reduction in STN size, and this led to a change in the overall shape of the STN, which appeared visibly narrower ventrally and dorsomedially (Fig. 8*D*). Three-dimensional quantification confirmed a 14% smaller diameter as well as a 38% reduction in volume in the cKO STN.

These results demonstrate that the targeted deletion of the *Vglut2/Slc17a6* gene in the STN causes substantial loss of neurons, primarily those of small cell area, which in turn likely has caused the alteration in the size and shape of the entire STN structure. This substantial alteration likely contributes to the motor and reward behavioral alterations observed. Further, the results obtained pinpoint *Vglut2/Slc17a6* gene expression levels as crucial for the structure and function of the STN.

## Discussion

The STN has gained wide attention based on its pivotal role in motor, limbic, and cognitive functions, and therapies based on electrical brain stimulation of the STN show a steady increase as a treatment of advanced PD and a range of neuropsychiatric disorders (Chabardès et al., 2013). Aberrant excitation from the STN not only contributes to the core symptoms of PD (bradykinesia, rigidity, tremor, and axial instability), but excessive excitation from the STN has also been proposed to contribute to the degeneration of midbrain DA neurons and may thus play a role in the etiology of the disease (Rodriguez et al., 1998; Olanow and Tatton, 1999; Menegas et al., 2015).

Although the so-called tripartite model of the STN dominates the current way of correlating the anatomy of the STN with its many functional roles, this model has recently been challenged (Keuken et al., 2012; Alkemade and Forstmann, 2014; Lambert et al., 2015). Interestingly, no molecular markers have been identified that could be used to delineate putative anatomical borders between the three main subdivisions of the STN; this lack of subdivision-selective markers contributes, in our mind, to the difficulty in firmly confirming or dismissing the tripartite model. Both Vglut2 and Pitx2 mRNAs localize within the mouse STN (Martin et al., 2004; Schweizer et al., 2014), but the current study shows that they are both distributed throughout the entire STN structure; neither Vglut2 or Pitx2 can thus serve as a selective marker for any specific aspect within the STN. We further conclude that whereas we previously referred to the *Pitx2/Vglut2* coexpressing neurons as a subpopulation of the STN (Schweizer et al., 2014), the demonstration that Pitx2 and Vglut2 mRNA overlap in almost 90% of all STN neurons now pinpoints that the *Pitx2/Vglut2* subpopulation represent the vast majority of the STN.

Given this broad distribution of Vglut2 and Pitx2 mRNAs throughout the STN, and the reduction of Vglut2 mRNA levels throughout the STN structure of the *Vglut2^f/f;Pitx2-Cre+^* cKO mice, a strong behavioral phenotype might not be surprising. The surprise, if any, rather lies in the seemingly opposing effects observed on motor and reward-related behavior. Although motorically hyperactive both vertically and horizontally, the cKO mice consume less sugar than control mice. Previous studies in rats selected for their low (LRA) or high (HRA) rearing activity have shown that HRA rats display elevated locomotor activity and collect rewards in the baited radial arms maze faster than LRA rats, but as they also make more working memory errors. These results suggested that higher reward consumption in hyperactive rodents is correlated with working memory failure (Görisch and Schwarting, 2006). Although the *Vglut2^f/f;Pitx2-Cre+^* cKO mice previously were shown to be strongly hyperactive both in terms of locomotion and rearing, they did not make more errors in the baited radial arm maze; neither reference or working memory errors were elevated in the cKO mice (Schweizer et al., 2014; Pupe et al., 2015). However, consistently throughout our natural reward-related tests (the baited radial arm and delay discounting tests as well as the sugar self-administration presented here), the *Vglut2^f/f;Pitx2-Cre+^* cKO mice consumed less than controls.

Based on these observed behavioral alterations, we argue that the motoric hyperactivity overrides the interest in palatable food. In efforts to unravel the biochemical correlates of such a behavioral phenotype, we identified a series of modifications that support this argument. First, a strong correlation between DA signaling in the NAcSh and reward-related behaviors is already well established (Everitt and Robbins, 2005), and dysfunction of the mesostriatal DA system is strongly implicated in disorders with a reward-related component, including addiction (Volkow et al., 2009), anorexia nervosa (Kontis and Theochari, 2012), and overeating (Wang et al., 2001; Stice et al., 2008). By now demonstrating that the targeting of *Vglut2/Slc17a6* has caused an increase in DA activity in the DStr (mainly motor) but decreased it in the NAc (mainly reward), opposite behavioral outcomes mediated by these two systems might indeed be achieved. The selective lack of interest in palatable food while showing normal levels of regular chow consumption is likely correlated with the biochemical alterations in the NAc. Increased DAT capacity suggests faster clearance of DA, whereas elevated levels of Dyn peptides might represent a biochemical indication for a dysphoric state in these animals.

Microinjection of the selective κ opioid receptor agonist DAMGO in the NAc of experimental animals has been shown to produce conditioned place aversion (Bals-Kubik et al., 1993), whereas an antagonist of these receptors causes an antidepressant effect (Shirayama and Chaki, 2006). The Dyn neuropeptides are mainly present in D1R-expressing cells of the NAcSh (Al-Hasani et al., 2015); although we did not observe D1R upregulation, the observed reduction in binding to D3R, which can oppose D1R activity, could have relieved D1R intracellular signaling to increase Dyn levels in the NAc (Schwartz et al., 1998). Thus, although elevated motor activity might be explained by the observed reduction of glutamatergic postsynaptic activity in the EP and SNr alongside the reduced DAT clearance in the DStr observed in the *Vglut2^f/f;Pitx2-Cre+^* cKO mice, the current findings of elevated DAT levels in the NAcSh as well as increased levels of both D2R and Dyn neuropeptides likely provide a biochemical correlate of the deficiency in the reward-collecting behavior demonstrated by the *Vglut2^f/f;Pitx2-Cre+^* cKO mice. Further, by unraveling the severe impact on the size and shape of the STN structure itself, we propose that slimming of the ventral (presumably associative) and part of the medial (limbic) aspects of the STN might cause the reduced dopaminergic activity in the NAc. Similar slimming of the STN structure has been observed upon ibotenic acid–induced STN lesioning, in which a substantial amount of STN cells are lost (Baunez et al., 1995, 2002), whereas targeted deletion of the *Vglut2/Slc17a6* gene in the midbrain has been shown to lead to reduced cell density (Fortin et al., 2012).

In the *Vglut2^f/f;Pitx2-Cre+^* cKO mice, expression of the *Vglut2/Slc17a6* gene is targeted from early embryogenesis onward. Having now verified that reduced Vglut2 mRNA levels occur throughout the STN structure, and also that the STN suffers from cell loss and structural modifications, we posit that this genetic intervention resembles to some extent a pharmacologic lesion model of the STN. Indeed, pharmacologic lesioning, as well as inactivation, of the STN increases motoric activity and has been shown more recently to affect motivated behavior, including consumption of palatable food (Baunez et al., 2002, 2005; Bezzina et al., 2008; Rouaud et al., 2010). Although pharmacologic lesioning and inactivation of the STN, as well as STN-DBS, in experimental animals show beneficial effects on motor behavior similar to STN-DBS in humans (Baunez and Gubellini, 2010), the results have been more variable and difficult to interpret in terms of motivated and reward-related behavior; this is likely because of different experimental paradigms and metabolic states of animals (e.g., food restriction) analyzed in the reported studies (Uslaner et al., 2007; Pratt et al., 2012).

Importantly, the *Vglut2/Slc17a6* “genetic lesion model” (represented by the *Vglut2^f/f;Pitx2-Cre+^* cKO mice) has a developmental onset that sets it apart from current pharmacologic lesion models. In the *Vglut2^f/f;Pitx2-Cre+^* cKO mice, there is a significant reduction in number of STN neurons in the adult animal, which in turn likely causes the alteration in STN shape. Addressing dysfunction in developmental processes, such as defects in proliferation or premature cell death, is beyond the scope of the present study. Further, STN neurons may not differentiate or migrate in a normal manner, and they may not even form functional connections in the absence of normal levels of *Vglut2/Slc17a6* gene expression.

The impact of a developmentally induced genetic lesion likely has a substantial effect of overall circuitry. Therefore, identifying the exact nature of the correlation between the reduced *Vglut2/Slc17a6* gene expression levels, the altered shape of the STN, and the observed effects on the DA system is not straightforward. Although we can conclude that there is no direct innervation of either the ventral or dorsal striatum by the *Pitx2/Vglut2* coexpressing STN neurons that might be directly affected by the targeted deletion of *Vglut2/Slc17a6*, we have established that both the EP and SNr are innervated by these STN neurons, and that the glutamatergic input into these targets is reduced in the *Vglut2^f/f;Pitx2-Cre+^* cKO mice. The EP is the main projection target in the STN motor loop, whereas the SNr also mediates associative functions (Benarroch, 2008), and we therefore hypothesize that the observed alterations in reward-related behavior might be connected more specifically to the STN-SNr pathway. Although STN neurons generally are excitatory in nature, high-frequency stimulation of the STN leads to a reduced drive for burst firing in the SNr (Shen and Johnson, 2008) and can, through endocannabinoid mechanisms, attenuate the GABAergic innervation of the SNc through the SNr (Freestone et al., 2015). The reduction in glutamatergic transmission in the STN of *Vglut2^f/f;Pitx2-Cre+^* cKO could therefore have led to a developmental alteration in the excitation/inhibition balance within the STN-SNr-SNc axis, presumably via the structurally disturbed ventral and part of the medial aspects of the STN.

Developmental effects might make it difficult to pinpoint additional circuitry components in the STN-EP/GP/SNr-striatum loop unraveled here. However, targeting the *Vglut2/Slc17a6* gene bears a physiological relevance that is not matched by toxin-induced models. Null mutations of the *Vglut2/Slc17a6* gene in mice are lethal directly after birth because of disturbance of the pre-Bötzinger respiratory central pattern generator (Wallén-Mackenzie et al., 2006), and the same is likely true for humans. However, some rare genetic variants of the human *VGLUT2/SLC17A6* gene have been identified in schizophrenia and severe alcoholism (Flatscher-Bader et al., 2008; Shen et al., 2010; Comasco et al., 2014). In the *Vglut2^f/f;Pitx2-Cre+^* cKO mice, expression levels of the *Vglut2/Slc17a6* gene are merely blunted, but not eliminated, and therefore effects may be exposed that are of relevance to human conditions. Indeed, we show that the blunting of *Vglut2/Slc17a6* gene expression levels selectively within the STN of mice causes opposite biochemical modifications in the dorsal and ventral striatal DA systems, followed by behavioral hyperactivity and decreased interest in palatable food, possibly due to a dysphoric state. We tentatively propose that genetic variants of the human *VGLUT2/SLC17A6* gene might be correlated with similar modifications. Further, by demonstrating an association between reduced Vglut2 mRNA levels in the STN and reduced STN size in mice, brain imaging analysis of STN size in human individuals might serve a purpose in future clinical investigations of human brain disorders implicating the STN, and where STN-based therapies are implemented already, such as PD and obsessive compulsive disorder, as well as for addiction, eating disorders and obesity, disorders proposed to benefit from STN-based therapy.

## Synthesis

Reviewing Editor: Thomas McHugh, RIKEN Brain Science Institute<br />Decisions are customarily a result of the Reviewing Editor and the peer reviewers coming together and discussing their recommendations until a consensus is reached. When revisions are invited, a fact-based synthesis statement explaining their decision and outlining what is needed to prepare a revision will be listed below. For some manuscripts transferred from JNeurosci, a decision is made to accept the manuscript without peer review, and a brief statement to this effect will follow.

The authors' changes in the text and response to the previous reviewers comments are sufficient to warrant publication with no further review.
